# Bacterial RNA Polymerase-DNA Interaction—The Driving Force of Gene Expression and the Target for Drug Action

**DOI:** 10.3389/fmolb.2016.00073

**Published:** 2016-11-09

**Authors:** Jookyung Lee, Sergei Borukhov

**Affiliations:** Department of Cell Biology, Rowan University School of Osteopathic MedicineStratford, NJ, USA

**Keywords:** RNA polymerase, transcription, promoter recognition, transcription initiation, transcription factors

## Abstract

DNA-dependent multisubunit RNA polymerase (RNAP) is the key enzyme of gene expression and a target of regulation in all kingdoms of life. It is a complex multifunctional molecular machine which, unlike other DNA-binding proteins, engages in extensive and dynamic interactions (both specific and nonspecific) with DNA, and maintains them over a distance. These interactions are controlled by DNA sequences, DNA topology, and a host of regulatory factors. Here, we summarize key recent structural and biochemical studies that elucidate the fine details of RNAP-DNA interactions during initiation. The findings of these studies help unravel the molecular mechanisms of promoter recognition and open complex formation, initiation of transcript synthesis and promoter escape. We also discuss most current advances in the studies of drugs that specifically target RNAP-DNA interactions during transcription initiation and elongation.

## Introduction

Bacterial multisubunit DNA-dependent RNA polymerase (RNAP) is the key enzyme of gene expression and a target of regulation. It is responsible for the synthesis of all RNAs in the cell using ribonucleoside triphosphates (NTPs) substrates. The core enzyme consists of five evolutionarily conserved subunits (α_2_ββ′ω) with a total molecular weight of ~380 kDa (Borukhov and Nudler, 2008). Although catalytically active, the core enzyme alone is unable to recognize specific promoter sequences, or melt the DNA and initiate transcription. For this, it associates with one of several specificity factors, σ (20~70 kDa), to form RNAP holoenzyme (α_2_ββ′ωσ) (Murakami and Darst, 2003; Decker and Hinton, 2013). In all bacterial species, most housekeeping genes are transcribed by holoenzyme of one major sigma factor, such as σ70 in *E. coli* or σA in *Thermus thermophilus*. Binding of alternative σ factors generates multiple forms of holoenzyme that can utilize different classes of promoters under various growth conditions and in response to environmental cues. The number of σ factors in different bacterial species varies widely from 1 in *Mycoplasma genitalium* to 7 in *E. coli* to 63 in *Streptomyces coelicolor*. Many regulatory factors besides σ can modulate RNAP's ability to recognize promoters and initiate transcription, modify its enzymatic functions and properties (Gruber and Gross, 2003).

### Overview of RNAP structure

In the last 16 years, a wealth of structural information on bacterial RNAPs core and holoenzymes was made available. Initially, the high resolution (2.5~4.5 Å) X-ray crystal structures of RNAP and RNAP complexes with nucleic acids, regulatory factors, and small-molecule inhibitors were obtained using thermophilic organisms, *T. thermophilus* (*Tth*) and *T. aquaticus* (*Taq*). Beginning in 2013, high-resolution structures of *E. coil* (*Eco*) RNAP holoenzyme and its complexes with nucleic acid and inhibitors began to emerge from several groups. Altogether more than 24 high resolution structures of RNAP/RNAP complexes to date have been deposited to database (Zhang et al., 2012, 2014; Bae et al., 2013, 2015a,b), (Bae et al., 2015c; Molodtsov et al., 2013, 2015; Murakami, 2013; Sarkar et al., 2013), (Zuo et al., 2013; Basu et al., 2014; Degen et al., 2014; Feng et al., 2015, 2016; Liu et al., 2015, 2016; Yang et al., 2015b; Zuo and Steitz, 2015). These include: the structures of *Taq* core; *Eco* core with transcription factor, RapA; *Taq, Tth*, and *Eco* holoenzymes and open promoter complexes; *Tth* ternary elongation complexes with and without transcription factors (Gfh1, GreA); *Taq* and *Tth* RNAP complexes with small-molecule inhibitors and antibiotics. Structural data compilation was also aided by high-resolution structures (1.8–2.9 Å) of sub-domains of *Eco* RNAP subunits β′, α, and σ, and their complexes with DNA and regulatory factors. For a comprehensive list of currently available bacterial RNAP structures, see the recent review (Murakami, 2015). Together with information gained from a wide range of biochemical, biophysical, and genetic studies, these data refine our understanding of bacterial RNAP structure-function and provide a broad view of transcription process and its regulation.

**Figure 1:**
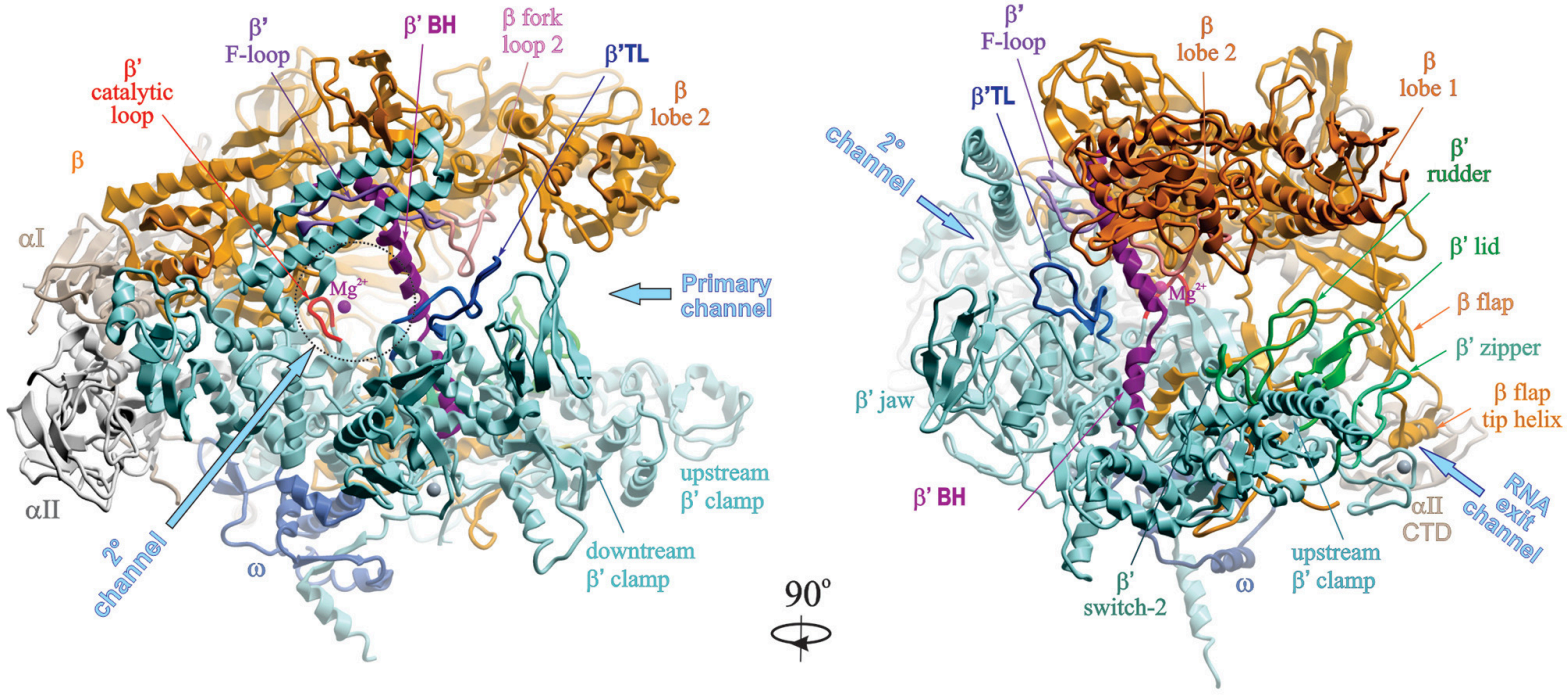
**Structural overview of RNAP core**. Structure of *Taq* RNAP core (PDB:1HQM; Zhang et al., 1999) shown in ribbons in two rotational views, using Molsoft ICM Pro program. Left panel, 2° channel view; right panel, main channel view. The structure is represented as colored ribbons (αI, olive; αII, light gray; β, yellow; β′, cyan; ω, blue) with important domains and structural elements indicated. The directions of primary, secondary and RNA exit channels are indicated by large arrows. Mg^2+^ ion is shown as a small magenta sphere. The structures of β′ trigger loop (TL), β′ rudder, β′ lid, β′ zipper, and β′ switch-2 regions are modeled using the structure of *Tth* holoenzyme (PDB: 1IW7; Vassylyev et al., 2002). The *Tth* β′ nonconserved domain (NCD1, G164-S449) is not shown.

The overall structure of a bacterial RNAP core enzyme resembles a crab claw, with the two clamps representing β and β′ subunits (Figure 1). The clamps are joined at the base by the N-terminal domains of σ-dimer (αNTDs) serving as a platform for RNAP assembly. σI-NTD and αII-NTD contact mostly β and β′ subunits, respectively. The C-terminal domains of α-dimer (αCTD), each tethered to NTD through a flexible linker, project out from the side of RNAP facing upstream DNA. The large internal cleft between β and β′ clamps is partitioned into the main “primary channel” that accommodates downstream dsDNA and RNA-DNA hybrid; the “secondary channel,” which serves as the site for NTP entry; and the “RNA exit channel” which is involved in RNA/DNA hybrid strand separation and interactions with RNA hairpins during pausing and termination. The active center is located on the back wall of the primary channel, at the center of the claw, where the catalytic loop with three aspartates holding essential Mg^2+^ ion resides. A long α-helical “bridge” (bridge helix, BH) connecting the β and β′ clamps, the two flexible α-helices of the “trigger” loop (TL), and an extended loop (F-loop), together with the catalytic loop, comprise the active center (reviewed in Nudler, 2009). The ω subunit is bound near the β′ C-terminus at the bottom pincer, serving as a β′ chaperone.

**Figure 2:**
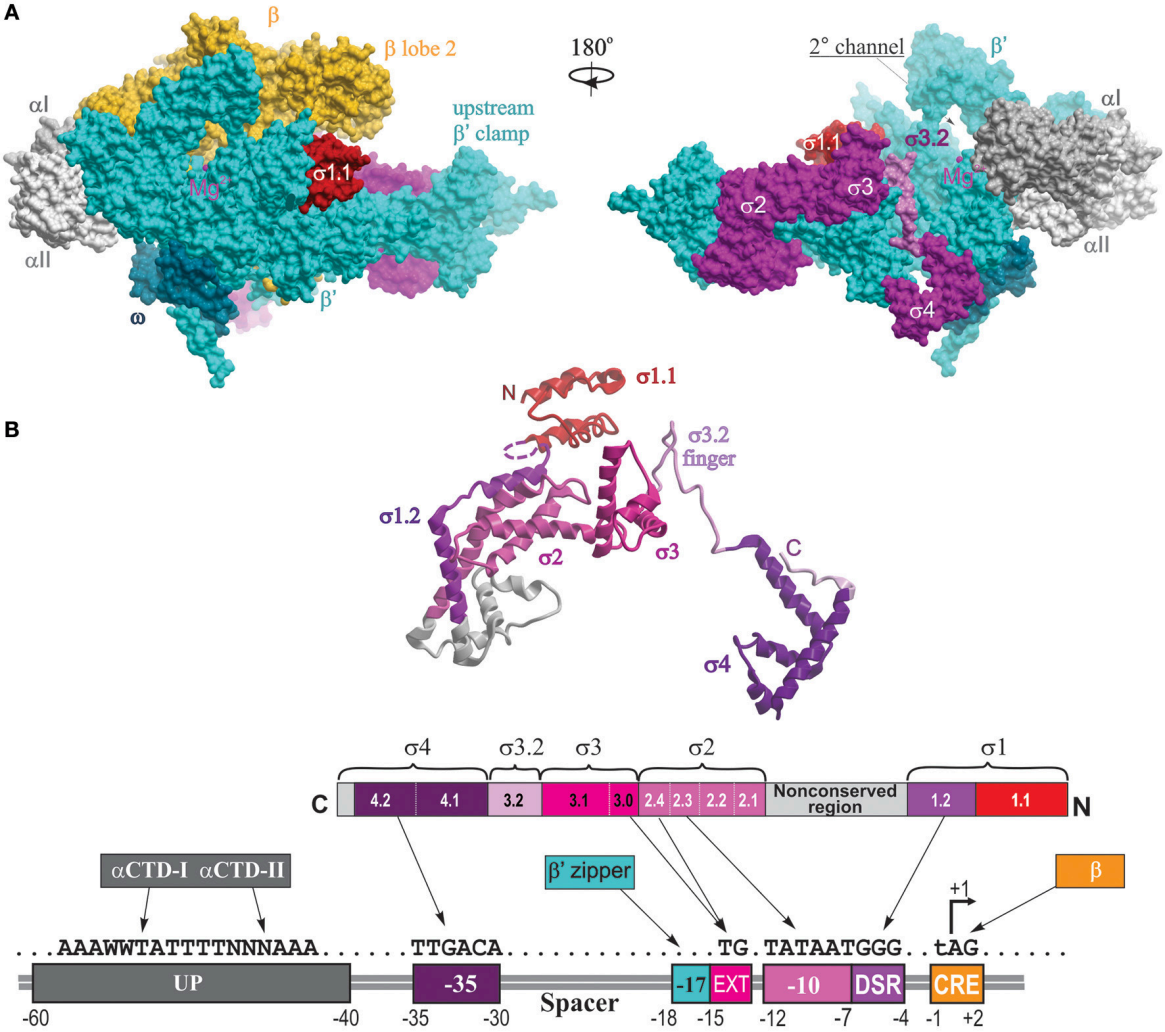
**Structural overview of RNAP holoenzyme and σ-DNA interactions. (A)** The structure of *Tth* σ^A^-holoenzyme is shown in molecular surface views with color coding as follows: αI, slate gray; αII, light gray; β, yellow; β′, cyan, σ, magenta, ω, dark cyan. Locations of conserved σ domains are indicated. The catalytic Mg^2+^ ion is shown as a small magenta sphere. The N-terminal domain of *Eco* σ^70^ carrying region 1.1 is modeled from the structure of *Eco* holoenzyme (PDB: 4YG2, Murakami, 2013) and shown as a red surface. Left panel, 2° channel view (as in Figure 1); right panel is obtained by rotation of the left panel view by 180° around the vertical axis, with the β subunit removed to reveal the location of σ3.2 finger region (colored light magenta) and σ4 occupying the RNA exit channel. **(B)** The structural and functional organization of σ. Top, a ribbon view of σ^A^ from *Tth* holoenzyme structure (PDB: 1IW7; Vassylyev et al., 2002) shown on the right panel in **(A)**. Colored regions correspond to the evolutionarily conserved domains of σ as shown in the functional map of σ70 below. Bottom diagram, a linear representation of σ polypeptide with structural domains and conserved regions shown as numbered and color-coded boxes. Underneath is a diagram of DNA promoter regions showing interactions made by DNA-binding domains of σ, αCTD β′ zipper, and CRE-binding β lobe-2 elements.

In the structure of σ70-holoenzyme, the bulk of the σ subunit (domains σ1–σ3) is bound on the core surface at the entrance to the major cleft, except for the linker connecting σ domains 3 and 4 (σ3–4 linker containing conserved region σ3.2), which threads through the primary channel, reaches the catalytic pocket with its hairpin loop (σ finger), and comes out from the RNA exit channel, almost completely blocking it (Figure 2). The rest of σ is wedged between the β and β′ clamps at the upstream side of the core enzyme, creating a wall that partially blocks the opening of the primary channel. Transition from core to holoenzyme is accompanied by partial closing of the β,β′ clamps by ~5 Å and movement of the flap domain (tip helix) induced by σ4 by ~12 Å (Vassylyev et al., 2002). The σ2, σ3, and σ4 domains are optimally positioned to contact the −10, extended −10, and −35 elements of the promoter DNA, respectively. In the crystal structures of *Eco* holoenzyme, consistent with previous biophysical studies (Mekler et al., 2002), σ region 1.1 is located in the downstream dsDNA binding region, blocking the access to DNA (Bae et al., 2013; Murakami, 2013). This location of σ1.1 explains why nonspecific transcription initiation by σ70-holoenzyme at promoterless DNA sequences is very low (Shorenstein and Losick, 1973). The σ-core interface is extensive with multiple cooperative contacts (Sharp et al., 1999; Gruber et al., 2001; Murakami and Darst, 2003), explaining the high stability of the σ-core association (*K*_*D*_ ~0.3 nM; Maeda et al., 2000). However, most of these contacts appear to be relatively weak (Vassylyev et al., 2002; Borukhov and Nudler, 2003), which allows alternate σ factors to successfully compete for binding to core. The conserved regions 2.1 and 2.2 of s2 make the most stable contacts with the upstream β′ clamp helices—the major s docking site (Figure 2). σ4 interacts with the β-flap domain, with the C-terminus of σ contacting the β-flap tip. In the presence of specific activators, σ4 also interacts with σI-CTD. In the recent structure of σ^S^-initation complex, σ^S^ regions from 1.2 to 4.2 display the same fold as σ^70^, including the linker 3.2 that inserts into the active site pocket (Liu et al., 2016). σ^S^ lacks the non-conserved domain present in σ^70^, which may explain its lower binding affinity to core (*K*_*D*_ ~4 nM) (Maeda et al., 2000).

### Overview of the transcription cycle

**Figure 3:**
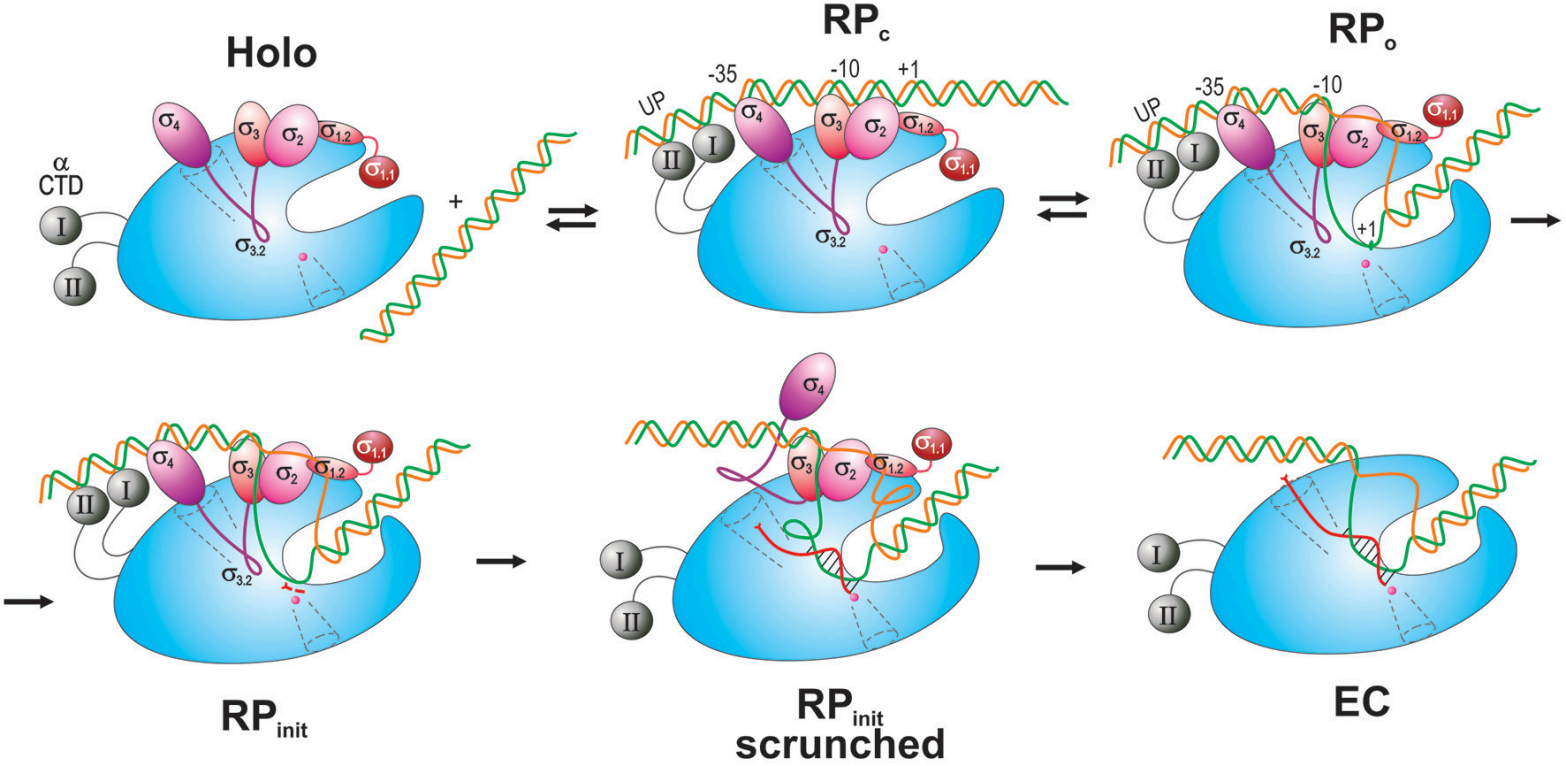
**Schematic overview of the main steps in transcription initiation**. RNAP is shown as a blue oval with the main channel cleft. αCTD is depicted as gray spheres connected to RNAP by flexible linkers (curved lines). σ domains are represented as colored ovals, except for σ3.2 which is drawn as a magenta curvy line connecting σ4 and σ3. The DNA *t*-strand and *nt*-strand are colored green and orange, respectively. The nascent RNA is shown as a curvy red line in a scrunched RPinit and in an EC with the DNA-hybridized section indicated. The scrunched part of the DNA bubble is shown as loops in RPinit scrunched. Two initiating NTPs are depicted as short red segments near the catalytic site in RPinit. The RNA exit channel and 2° channel are shown as funnels in dotted line. The catalytic Mg^2+^ ion is shown as small magenta sphere at the end of the 2° channel.

Transcription process consists of three major stages: initiation, elongation, and termination. In bacteria, initiation occurs through five steps (Figure 3; reviewed in Murakami and Darst, 2003; Saecker et al., 2011).

*First*, RNAP core enzyme, composed of five subunits (α_2_ββ′ω), binds one of several specificity factors, σ (such as σ^70^ for transcription of housekeeping genes in *Escherichia coli*) to form holoenzyme (Eσ^70^). *Second*, Eσ^70^ recognizes and binds promoter DNA, a pair of conserved hexameric sequences present at positions −35 and −10 relative to the transcription start site (TSS), where it forms a closed promoter complex (RPc). Sequences immediately upstream and downstream of the −10 element including “^−15^TG^−14^ extended −10” (Keilty and Rosenberg, 1987), “−15 enhancer” (or “−17/−18 zipper-binding”; Liu et al., 2004; Yuzenkova et al., 2011) and “^−6^GGGA^−3^ discriminator” (Feklistov et al., 2006; Haugen et al., 2006) regions, and A/T-rich regions upstream of the −35 element [“UP-element” at −45; −65 (Ross et al., 1993)], also contribute to specific recognition by the Eσ^70^ (reviewed in Decker and Hinton, 2013; Feklístov et al., 2014). *Third*, RPc undergoes a series of conformational changes (isomerization) through several transition states (such as intermediate complex, RP_i_), to form an open promoter complex (RPo). Isomerization results in unwinding of DNA duplex around the −10 region (typically, between nt −11 and +2- +4) and creates a 12–15 nt long transcription bubble, a hallmark of RPo. *Fourth*, in the presence of rNTPs, RPo converts to an initial transcribing complex (RP_init_), forms the first phosphodiester bond between rNTPs positioned at +1 and +2 sites and then begins the RNA synthesis. During the synthesis beyond dinucleotide, RP_init_ undergoes “scrunching” whereby the downstream DNA (from +2 to +15) is pulled into the enzyme to be transcribed, resulting in bubble expansion (up to ~25 nt), while the upstream DNA-RNAP contacts remain intact (Kapanidis et al., 2006; Revyakin et al., 2006). At the same time, growth of nascent RNA beyond 6-mer is obstructed by the presence of σ3–4 linker in the RNA exit channel (Basu et al., 2014; Bae et al., 2015b). Biochemical data suggest that stress associated with DNA scrunching, and more importantly the steric clash between RNA 5′-end and σ3–4 (σ finger), together cause RNAP to repetitively synthesize and release short RNAs without leaving the promoter (abortive initiation; Sen et al., 1998; Murakami et al., 2002b; Kulbachinskiy and Mustaev, 2006; Samanta and Martin, 2013; Winkelman et al., 2016b). *In the final* step, the enzyme synthesizes an RNA of a critical length (typically 11–15 nt, of which ~9 nt are in the transcription bubble as RNA–DNA hybrid), removes the exit channel blockage, and escapes from promoter, entering the elongation stage of transcription. During this transition, RNAP undergoes global conformational change, which leads to the loss of RNAP-promoter DNA contacts, gradual σ dissociation, and formation of a highly stable and processive ternary elongation complex (EC) (Murakami and Darst, 2003).

Throughout elongation stage, the size of the transcription bubble in the EC remains constant at ~12 ± 1 nt, and the size of RNA/DNA hybrid is maintained at ~9–10 bp (Svetlov and Nudler, 2009; Kireeva et al., 2010). Elongating RNAP can transcribe DNA over long distances (>10,000 bp) without dissociation and release of RNA product. However, elongation does not proceed at a uniform rate; monotonous movement of RNAP can be interrupted by various roadblocks imposed by certain DNA sequences, DNA topology, lesions in transcribed DNA template, RNA secondary structures, DNA-binding proteins, DNA replication and repair machineries, ribosomes, other transcription complexes, RNAP-binding transcription factors (including σ70 that can be recruited back to EC upon encountering promoter-like sequences; Goldman et al., 2015; Sengupta et al., 2015), and small-molecule effectors (ppGpp) (Landick, 2006; Perdue and Roberts, 2011; Nudler, 2012; Imashimizu et al., 2014; Belogurov and Artsimovitch, 2015; Kamarthapu et al., 2016). Eventually, RNAP encounters a termination signal—a 20–35 nt-long G/C-rich RNA sequence of dyad symmetry that forms a hairpin structure immediately followed by a 7–9 nt-long stretch of Us (Yarnell and Roberts, 1999). During termination, RNAP releases the nascent transcript and dissociates from the DNA template, after which it can rebind a σ factor and start a new round of transcription. Under certain conditions, transcription termination can also be induced by termination factors ρ and Mfd (Roberts and Park, 2004; Kriner et al., 2016).

In this review, we discuss recent findings that elucidate molecular details of RNAP-DNA interactions during initiation the transcription cycle. Specifically, we will describe most current advances in the structural and biochemical studies of the molecular mechanisms underlying promoter recognition and RPo formation, activation of initiation, and promoter escape. Finally, we will review the mechanisms of action of known antibacterial drugs that specifically target RNAP.

## Molecular details of transcription initiation and its activation

### Structure of holoenzyme-promoter DNA complexes

#### Promoter recognition and binding: closed promoter complex (RP_c_)

Currently, there are no high resolution structure of the RP_C_, but a model of *Taq* RP_c_ based on the existing structural, biochemical, biophysical, and genetic data has been proposed (Murakami et al., 2002a; Murakami and Darst, 2003). In the model, promoter dsDNA rests on the outer surface of the RNAP main channel, bound mostly by σ (Figure 3). RNAP contacts with −10, extended −10, and −35 elements of the promoter are established by σ2, σ3, and σ4 (regions 2.2–2.4, 3.0, and 4.2, respectively) through polar and van der Waals contacts. Additionally, residues of two αCTD helix–hairpin–helix motifs (*Eco R265, N294*, and *K298*) may contact A/T-rich sequences in the minor groove of the UP element at positions from −40 to −60 and up to −90 (Ross et al., 2001; Benoff et al., 2002). These weak but specific UP/αCTD interactions contribute to RPc formation (Haugen et al., 2008). However, with the exception of the −35 element/σ4 and the −12 bp of −10 element/σ2 contacts, other upstream dsDNA-RNAP interactions are mostly non-specific and weak, which makes RP_c_ intrinsically unstable. Nonetheless, these interactions may provide initial promoter recognition and increase its occupancy by RNAP. They may also induce local distortion in DNA structure facilitating subsequent steps in transcription initiation: DNA melting, strand separation and template strand insertion into the active site cleft. For instance, the RNAP-bound DNA in the RPc appears to be bent or kinked at three places: around −25, to accommodate variable spacer length, at −35, induced by insertion of s4 helix-turn-helix motif into the major groove, and at −45, induced by αCTD-DNA minor groove interactions (Benoff et al., 2002). The DNA bending at −35 aids in the proper binding of upstream DNA by αCTD and upstream transcription activators. Additionally, recent structural and biochemical data on RPo (see below) implicate conserved residues of σ3 region 3.0 (*Tth* H278 and R274) and β′ zipper (*Tth* Y34 and R35, T36, and L37) in the recognition of non-canonical −17/−18 “Z-element” which contribute to promoter recognition and binding in RPc (Yuzenkova et al., 2011; Bae et al., 2015b). Notably, σ1.1 blocks the access to downstream (ds and ss) DNA in the main channel by binding with its negatively charged face (mimicking DNA) to the basic surface of the β lobe-2 and the downstream β′ clamp (Murakami, 2013). However, the opposite, positively charged face of σ1.1 is positioned to interact with the downstream DNA which may further stabilize RPc. Subsequent steps leading to displacement of σ1.1 by downstream DNA during transition to RPo are poorly understood, but are thought to involve β′-clamp opening triggered by upstream promoter DNA binding and initial DNA unwinding around −11 (see below).

Recently an alternative view on initial promoter recognition and RPc formation was proposed based on structural and biochemical data (Feklistov and Darst, 2011; Zhang et al., 2012; reviewed in Hook-Barnard and Hinton, 2007; Decker and Hinton, 2013; Feklistov, 2013). In this view, except for direct RNAP recruitment by transcription activators that provide sequence-specific DNA recognition, the upstream DNA-RNAP interactions (involving UP and −35 elements) play only an auxiliary role in RPc formation. Instead, initial promoter binding/recognition is accomplished through (i) indirect readout of DNA shape (a distinctive conformational patterns of stacked bases in dsDNA) by RNAP, and (ii) by direct readout of an indispensable −10 element by σ2-specific interaction with two flipped-out consensus nucleotides, −11(A) and −7(T), of nt-strand DNA (see below). These two views on the mechanism of promoter recognition are not mutually exclusive and could be eventually addressed when the structure of RPc becomes available.

#### Advances in structural studies of open promoter complex (RP_o_)

**Figure 4:**
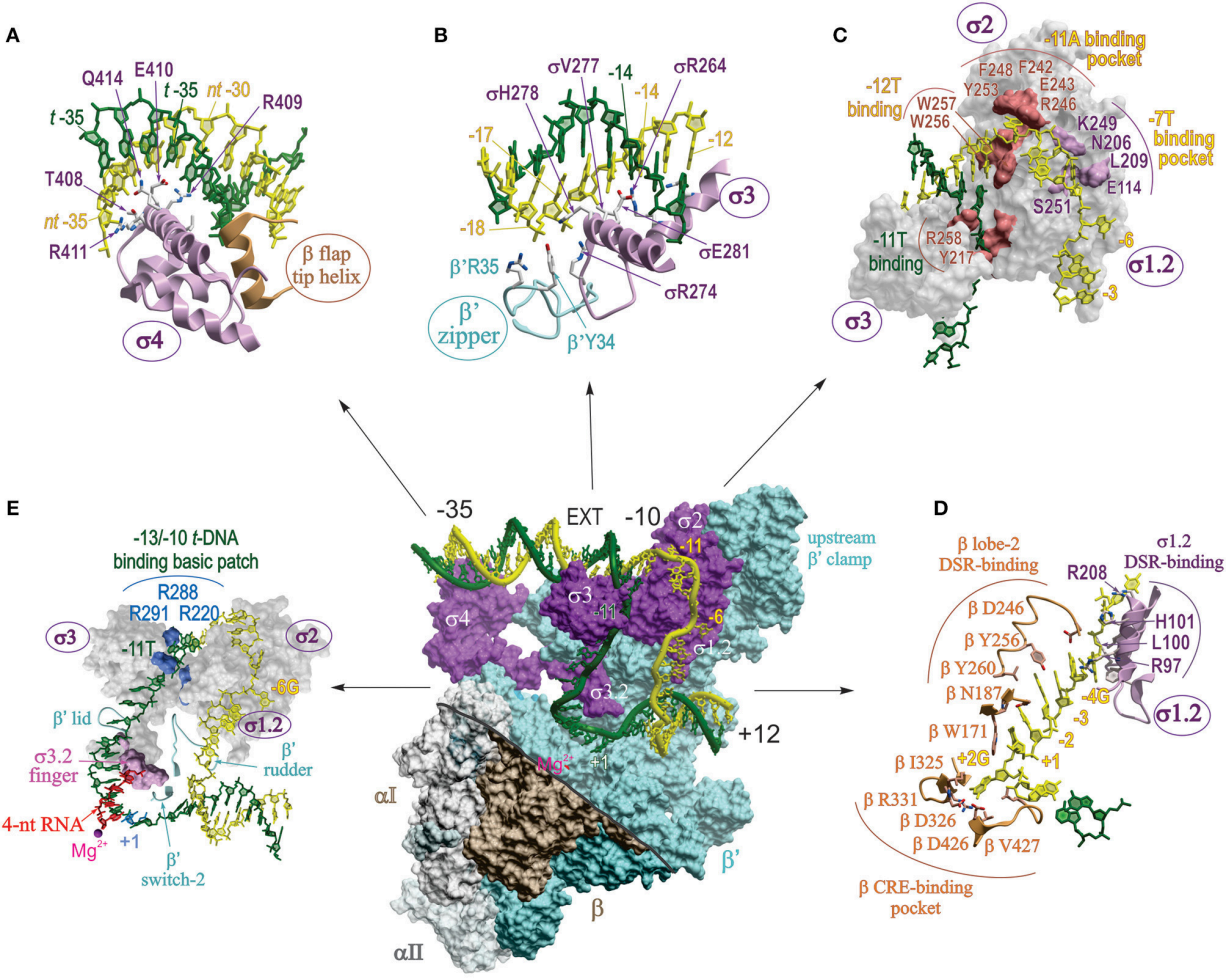
**Detailed map of protein-nucleic acid interactions in RPo**. Central panel shows the surface view of RPo structure of *Taq* σ^A^-holoenzyme with full bubble promoter DNA (endpoints −36; +12) (PDB: 4XLN; Bae et al., 2015b). The color coding is the same as in Figure 2, except for β which is shown in brown. The displaced σ1.1 domain and the β′-NCD1 are not shown. Part of the β structure is removed (above of the thick gray line) to reveal the inside of the main channel and to show the interactions of σ and β′ with transcription bubble and ds DNA. DNA is shown as a worm in stick representation with *t* and *nt* strands colored dark green and yellow, respectively, with the main promoter elements indicated. EXT, extended −10. The small magenta ball indicates the position of catalytic Mg^2+^. **(A–E)** Zoomed-in detailed views of different sections of the central panel with key interacting residues and nucleotide positions indicated as sticks **(A,B,D)** or as surfaces **(C**,**E)**. Interactions of **(A)** σ4 with the -35 DNA element; **(B)** β′ zipper and σ3 with −18/−17 and the extended −10 elements; **(C)** σ3 and σ2 with −10 element; **(D)** σ1.2 and β lobe-2 with DSR and CRE of the nt-strand DNA; **(E)** σ1.2–σ3.2 domains and β′ rudder, lid and switch-2 elements with the t-strand DNA spanning −11 to +1. Panel **(E)** highlights the interactions between basic patch residues of σ3 with the −11T at the upstream edge of transcription bubble, and the steric clash between the 4 nt-long RNA 5′ terminus and σ3.2 finger.

In the last 3 years several high resolution structures of *Tth, Taq*, and *Eco* RPo with different DNA scaffolds have been solved. These include upstream fork and downstream fork promoter DNA (Murakami et al., 2002a; Zhang et al., 2012, 2014; Basu et al., 2014), and complete transcription bubble promoter template with upstream and downstream dsDNA (Bae et al., 2015b; Zuo and Steitz, 2015; Liu et al., 2016). The structures of RPo correlate well and complement each other. Taken together, they reveal the positions of ds and ssDNA (from −36 to +12) in the complex and the key residues of RNAP that make critical interactions with DNA and RNA. Unlike RPc, in RPo both strands of downstream dsDNA up to +12 are fully enclosed inside the RNAP main channel (Figure 4). In RPo, RNAP makes tight contacts with DNA from −36 to −30 and −18 to +9, in agreement with DNA footprinting and crosslinking data (Ross and Gourse, 2009; Winkelman et al., 2015). The RNAP interactions with the upstream portion of ds DNA (from −36 to −12) are similar to that shown in RPc model, however, at −13/−12 the DNA bends sharply by ~90° toward the RNAP. At position −11, the t- and nt-strands separate, and enter different paths for ~13 downstream nucleotides until they form dsDNA at position +3, thus creating the “transcription bubble.”

##### Upstream DNA (−35, −17/−18, and extended −10 elements)

In two RPo structures that contain a full bubble DNA (Bae et al., 2015b; Zuo and Steitz, 2015), contacts made at −35 region are the same as in isolated σ4 domain/−35 element (Figure 4A) (Campbell et al., 2002). As proposed for RPc, RPo structure demonstrates that duplex DNA upstream of the −10 element (−18 to −12) makes functionally important contacts with conserved residues of β′ zipper, σ3, and σ2 (Figure 4B), mostly through phosphate backbone of the nt-strand (Yuzenkova et al., 2011; Bae et al., 2015b; Feng et al., 2016). These contacts were not visible in the low resolution structures of RNAP with nucleic acids. The sequence specific recognition of extended −10 element (−15T:A, −14G:C) by conserved residues of σ2/σ3, E281 (*Eco E458*), R264 (*R441*), and V277 (*V454*), stabilizes RPo and can substitute for a poor or absent −35 element (Keilty and Rosenberg, 1987). Mutational analysis shows that all three residues are essential for promoter recognition (Daniels et al., 1990).

##### −10 element

The initial DNA melting starts at the A:T bp at position −11 (Chen and Helmann, 1997; Lim et al., 2001), when −11A flips out from the duplex DNA, and continues downstream to +1. Two groups of aromatic residues are involved in the initial stages of DNA melting (Figure 4C). First, W256 and W257 forming a chair-like structure interact with base-paired −12T at the upstream edge of the bubble replacing the flipped-out −11A. The second group of aromatic residues Y253, F242, and F248, together with two polar residues R246, E243 form a pocket that captures the flipped-out −11A base. Additionally, in the context of a true promoter, the −11T on the t-strand orphaned by the flipping of −11A, may be stabilized by stacking interaction with another highly conserved aromatic residue Y217 (Bae et al., 2015b). Neither the W256A nor Y217A substitution affected the promoter binding, but rather decreased the rate of RPc → RPo isomerization. Based on this, it is proposed that these residues maintain the ds-ss junction at the upstream edge of the bubble, preventing bubble collapse and RPo dissociation. In another structure of RPo with full bubble DNA and activator protein, R258 (*R436*) stacks on −12A of t-DNA, facilitating flipping of the t-strand −11 base (Feng et al., 2016). The second canonical nucleotide of the −10 element, −7T of nt-strand, is flipped out and captured in a pocket made of five σ2 and σ1.2 residues: E114, N206, L209, K249, and S251 (*Eco E116, N383, R385, L386, S428*; Figure 4C), all of which are functionally important (Zhang et al., 2012). Other nucleotides in −10 element make mostly nonspecific contacts with σ2.

##### Discriminator

The “discriminator” (DSR) region of nt-strand (consensus sequence −6G, −5G, −4G) interacts with eight σ1.2 residues, of which *Tth* L100 (*Eco M102*) and *Tth* H101 (*Eco R103*) provide the most functionally important contacts (Figure 4D). The purine-rich DSR contributes to the high stability of RPo, whereas pyrimidine-rich sequence in this region destabilizes it (Haugen et al., 2006). This is due to change in the interaction made by the key nucleotides of DSR (−5) with σ; when it is G, it forms and maintains an ordered, stacked conformation of the nt-strand, but when it is C, it flips and is captured by a pocket in σ2, resulting in unstacking and compaction of the downstream ssDNA. Importantly, the presence of C in the middle of DSR in rRNA promoters is also one of the determinants of the transcription start site (TSS) selection (Haugen et al., 2006; Winkelman et al., 2016b). The DSR–σ1.2 interaction is a major determinant of the susceptibility of rRNA and other promoters to negative regulation by ppGpp and DksA (Haugen et al., 2006).

##### CRE

Nucleotides at positions −3 to +2 on nt-strand constitute “core recognition element” (CRE) that interacts specifically with 10 residues of RNAP β-subunit. Six of these residues form a pocket that captures the flipped-out +2G of CRE at the downstream edge of the bubble, reminiscent of the capturing of flipped-out −11A by σ2. In the pocket, *Tth* βD326 (*Eco D446*) makes a hydrogen bond with +2G, which proved to be most critical (Zhang et al., 2012). Adjacent residue *Tth* βW171 (*W183*) unstacks the +1T away from the +2G, facilitating its placement into β pocket. In addition to stabilizing and maintaining transcription bubble in RPo, CRE-core interaction affects sequence-specific pausing, and determines TSS selection (Vvedenskaya et al., 2016; Winkelman et al., 2016b). Moreover, it is predicted that CRE-RNAP interactions will affect all stages of transcription where unwound transcription bubble is involved, e.g., slippage, abortive synthesis, promoter escape, factor dependent pausing, termination.

##### T-strand

A cluster of conserved, positively charged residues of σ2.4 and σ3.0 (*Taq* R288, and R291, R220) pulls the DNA t-strand from −13 to −10, bending it by 90° through electrostatic interaction with the phosphate backbone, into a groove formed by σ3 linker, β′ lid, and the β′ rudder (Figure 4E; Bae et al., 2015b; Zuo and Steitz, 2015). The t-strand DNA (−9 to −5) is then placed into the main channel between the active site wall, mostly of β, and s3.2 hairpin loop (σ finger), which participates in juxtaposing DNA +1 position to the catalytic center (Figure 4E). Simultaneously, the dsDNA downstream of +3 to +12 is brought inside the downstream DNA binding clamp between the β′ jaw, β lobe-2 and the downstream β′ clamp. The β′ switch-2, a small flexible loop residing in the upstream β′ clamp in the middle of the main channel cavity, controls the binding of the downstream part of the unwound DNA t-strand in the active site cleft. β′ switch-2 functions as a hinge mediating opening and closing of the β′ clamp, and plays a critical role in downstream propagation of transcription bubble during formation of RPo (Mukhopadhyay et al., 2008; Belogurov et al., 2009; Bae et al., 2015b).

##### Role of σ3.2 finger in RP_init_

Recent structural studies showed that during initial transcript synthesis, σ3.2 loop physically occupies the path of nascent RNA and sterically blocks its extension beyond 4~5 nucleotides (Zhang et al., 2012; Basu et al., 2014; Bae et al., 2015b; Zuo and Steitz, 2015). Consistent with its position in the structure of RPo and RP_init_, biochemical data show that σ3.2 finger positively affects the binding of the first two initaiting NTPs, abortive RNA synthesis, and promoter escape (Murakami et al., 2002b; Kulbachinskiy and Mustaev, 2006). More recent studies revealed that σ3.2 contributes to promoter opening (Morichaud et al., 2016), suppresses σ-dependent promoter proximal pausing, and accelerates σ dissociation during transition from initiation to elongation (Pupov et al., 2014).

##### Transcription start site selection (role of DNA scrunching in RPo)

Transcription typically initiates 7 or 8 bp downstream from the −10 element, with a strong bias for purine (R) over pyrimidine (Y) as the initiating nucleotide (+1 position) (Shultzaberger et al., 2007). To identify the determinants for TSS selection, Nickels and coworkers combined a high throughput sequencing (MASTER, “massively systematic transcript end readout”) with multiplexed site-specific RNAP-DNA crosslinking and X-ray structural analyses, to dissect and characterize the role of sequence variation within −6 to +4 positions of the promoter on TSS selection (Winkelman et al., 2016b). The studies identified DSR and CRE as sequence elements that significantly influence TSS selection. G-rich DSR (GGG) and +2G CRE shortens the distance between TSS and −10 element (6-/7-nt from the edge of −10 element), whereas pyrimidine-rich DSR (CCC) and lack of CRE shifts TSS further downstream (8-/9-nt from the edge of −10 element). Disrupting the DSR-σ1.2 and/or CRE-β pocket interactions results in downstream shift of TSS. The changes in TSS correlate with the corresponding shift in the downstream edge of the transcription bubble (in + or − direction), while the upstream edge of the transcription bubble (−10 element) remains constant, demonstrating TSS selection involves transcription-bubble expansion (“scrunching”) and transcription-bubble contraction (“anti-scrunching”; Vvedenskaya et al., 2015; Winkelman et al., 2016b).

Importantly, the unique features of ribosomal promoter sequence (short suboptimal 16 bp spacer, absence of extended −10, and the lack of interactions of DSR/σ1.2, and CRE/β pocket) lead to RPo pre-scrunching and downstream shift of TSS to an unusual position 9 nt from the −10 element. These features reduce abortive synthesis and facilitate promoter escape during initiation, contributing to the high transcriptional activity of rRNA promoters (Winkelman et al., 2016a). At the same time, they destabilize RPo and increase its sensitivity to initiating NTP concentrations, providing a mechanism for rapid down-regulation during starvation.

Besides the TSS region sequences, the negative DNA supercoiling that increases the size of the transcription bubble in RPo also causes downstream shift in the TSS position (Vvedenskaya et al., 2015). These results are consistent with biophysical data correlating bubble expansion with TSS selection (Robb et al., 2013).

##### Initiation of RNA synthesis

Structures of RP_init_ were obtained by stabilizing the crystal structures of RPo with RNA oligos complementary to t-strand in the bubble from positions −4 to +1. However, these structures do not reflect the natural state of nascent transcript and DNA during transcription initiation. Recently, more functionally relevant structural data were obtained for RP_init_ by soaking the crystals of RPo with two initiating substrates, ATP and a non-hydrolyzable anolog of CTP, CMPcPP, occupying *i* and *i*+1 sites, respectively. The structure revealed the location of initiating substrate, ATP, in the catalytic center. However, CMPcPP does not occupy the catalytically reactive site since the position of its α-phosphate and the second Mg^2+^ ion coordinated by its β- and γ-phosphates are too far for catalysis. Also, the trigger helix is partly disordered and does not interact with phosphates of the substrate. Therefore, it is proposed that this structure captures RPinit in a transient state where, *i*+1 nucleotide is in pre-catalytic conformation (Basu et al., 2014; Zhang et al., 2014; Zuo and Steitz, 2015). *De novo* synthesis of 6-nt long transcript *in crystallo* generated a structure of RPinit with RNA-DNA hybrid and the scrunched downstream dsDNA. In the structure, σ3.2 finger is displaced from its position near the active site by the RNA 5′ end, signaling the beginning of σ release from RNAP (Basu et al., 2014).

During scrunching, the pulled-in portions of t- and nt-strands must bulge out of the transcription bubble. Because the X-ray structures of the scrunched ssDNA in RPinit are disordered, their paths have been recently assessed by site-specific DNA-protein crosslinking (Winkelman et al., 2015), exploiting the unusual stability of RPinit formed at ribosomal rrnBP1 promoter (Borukhov et al., 1993). In RPinit containing 5-mer RNA, the nt-strand bulge is extruded through the space between lobe 1 and 2 of β clamp into solvent, whereas the t-strand bulge remains inside the RNAP main channel restricted by the β flap, β lid, β′ clamp, and σ3.2 finger. Mapping results indicate that the t-strand bulge moves toward RNA exit channel, but its exact position is unclear. Extension of RNA beyond 5–6 nt will lead to further bulge expansion resulting in stress build-up, which can be relieved by displacement of σ3.2 finger and/or by opening of the β flap and β′ clamp domains. Further stress accumulation may cause the t-strand bulge to extrude outside either through expansion of the RNA exit channel or through the space opened up between β lobe 1 and σ3. Eventually, the growing 5′ end of nascent RNA will occupy the exit channel displacing σ3 and 4, commencing promoter escape. Another way to relieve the stress caused by t-strand bulge expansion is to reverse the scrunching by releasing the abortive RNA products through the 2°-channel and repeat initiation (Kapanidis et al., 2006; Revyakin et al., 2006).

#### Activation of transcription initiation

Many bacterial promoters contain suboptimal sequences that require binding of specific factors for efficient transcription initiation. Various classes of activators act by facilitating RNAP recruitment to promoters and by accelerating isomerization steps in initiation pathway: RPc → RPi → RPo → RPinit → promoter escape (reviewed in Roy et al., 1998; Lee et al., 2012; Decker and Hinton, 2013). Below, we present two examples of transcription activation systems that have been recently characterized with structural, genetic, and biochemical studies.

#### Transcription activation by class II initiation factors: CAP/TAP

Two well-characterized classes of transcriptional activators (class I and II) act through simple RNAP recruitment to promoters with missing or inefficient core promoter elements. Class I activators, exemplified by *E. coli* CAP (catabolite activator protein) binding at the −61.5 DNA site upstream of the *lac* promoter, stimulate RPc formation through direct interactions with the RNAP αCTD. Class II activators, such as *E. coli* CAP binding at the −41.5 site overlapping the −35 element of *gal* promoter, facilitate formation of RPc and its isomerization into transcriptionally active RPo through multiple contacts (activation regions AR1–AR3) with RNAP αNTD, αCTD, and σ4 domains (Lawson et al., 2004). A structural model of an *E. coli* class I transcription activation complex of CAP-RPo on a modified *lac* promoter based on a low resolution electron microscopy data has been generated (Lawson et al., 2004; Hudson et al., 2009) providing information on CAP/DNA, αCTD-DNA, and αCTD-σ4 interactions.

Recently, a 4.4 Å-resolution crystal structure of class II transcription activation complex was reported. It shows *Tth* activator protein TAP, a homolog of *E. coli* CAP, in complex with *Tth* RPo assembled on a TAP-dependent *Tth crtB* promoter, with a full transcription bubble and a 4 nt-RNA primer (Feng et al., 2016). In the structure, *Tth* TAP homodimer is bound to DNA at position −41.5 from transcription start site. As expected based on their sequence homology, the structures of *Tth* TAP-DNA and *Eco* CAP-DNA are very similar. The structure of RPo is mostly unchanged in TAP-RPo, except that DNA upstream of −35 is slightly distorted by TAP, resulting in reduced interaction between −35 region and σ4. Not surprisingly, biochemical data indicate that specificity of −35 recognition by σ4 does not play a major role in Class II CAP-mediated activation (Rhodius et al., 1997). Yet, intriguingly, mutations of the two σ4 residues (R584, E585) contacting bases at −32 and −33 strongly inhibited RPo formation (Feng et al., 2016). Apparently, the few remaining specific interactions between σ4 and −35 still play an important role in TAP mediated activation.

##### Role of αCTD in activation

In TAP-RPo structure, the distal subunit of TAP homodimer interacts with one αCTD through an interface that includes ~8 pairs of partnering residues. This interaction (mediated by activation region AR4, a unique feature of TAP) is essential for RPc formation, as demonstrated by the loss of promoter binding following substitutions of AR4/αCTD interface residues. In addition, unlike *Eco* CAP-RPo, αCTD in TAP-RPo does not interact with DNA. DNA footprinting data show that *Tth* αCTD does not contribute to DNA binding irrespective of TAP or promoter sequences (Feng et al., 2016), indicating that αCTD is used by TAP only as an RNAP-tether. Indeed, CAP and TAP use different regions to contact αCTD (AR1 and AR4, respectively) that may play a different role in activation. Whereas *Eco* CAP-AR1/αCTD interaction serves to recruit RNAP to the promoter by DNA-bound CAP, TAP-AR4/αCTD interaction facilitates the association of free RNAP and TAP prior to promoter DNA binding. Because the RNAP-TAP binding constant (6 μM) is comparable to the intracellular RNAP concentration [>5 μM (Patrick et al., 2015)], it is proposed that in addition to classic recruitment pathway, TAP may activate transcription via a pre-recruitment pathway, similar to the mechanism of eukaryotic transcription activation.

##### The role of TAP-AR2 and -AR3 in activation

TAP-AR2 interacts with β flap domain while TAP-AR3 interacts with σ4 and the β-flap tip helix. Most of these interactions are mediated through polar contacts and salt bridges, and are conserved between *Eco* CAP and *Tth* TAP. Mutation of residues in the AR2/β-flap, AR3/β-flap tip, and AR3/σ4 interfaces lead to defects in transcription activation by TAP. Kinetic analysis indicate that similar to CAP (Niu et al., 1996; Rhodius et al., 1997; Rhodius and Busby, 2000), interactions of TAP AR2- and AR3- with RNAP accelerate the transition of RPc to RPo but do not play a significant role in the initial DNA binding (RPc formation).

From the observation that the RPo structure does not change upon interaction with TAP, it is inferred that the mechanism of TAP/CAP Class II activation entails sequential stabilization of intermediate complexes (between RPc and RPo) through simple contacts between ARs and RNAP without inducing any conformational perturbations in RNAP. It should be noted, however, that the reported TAP-RPo structure presents the complex in the final activated state, whereas the path to this state is poorly understood. An alternative to the “activation by adhesion” mechanism can be envisaged which entails allosteric/conformationl changes in the intermediate initiation complexes.

#### Transcription activation by RPo stabilization: CarD

Unlike RNAP of the model organism *Eco*, RNAPs of many bacteria form intrinsically unstable RPo even at consensus promoters, and require additional factors to stabilize RPo. One such factor is CarD, a global regulator which is an essential factor in *Mtb*. CarD is widely distributed in at least ten bacterial phyla, including Firmicutes, Cyanobacteria, Actinobacteria, and Deinococcus-Thermus (Bae et al., 2015a), but is absent in γ-Proteobacteria such as *Eco*. The structure and function of *Mtb* and *Tth* CarD have been characterized (Stallings et al., 2009; Gulten and Sacchettini, 2013; Srivastava et al., 2013), and the molecular mechanism of its action was recently proposed based on the 3-D structure of *Tth* CarD in complex with RPo assembled on consensus promoter DNA with full transcription bubble (Bae et al., 2015a).

CarD consists of N-terminal, RNAP-interacting domain (CarD-RID) and α helical C-terminal domain (CarD-CTD). In CarD-RPo structure, CarD-RID binds RNAP β lobe-1 domain, orienting CarD-CTD toward the upstream ds/ss junction of the transcription bubble near the −10 element. One of the α-helices of CarD-CTD inserts a conserved W86 into the DNA minor groove near positions −12/−13, and acts as a wedge to maintain the distorted conformation of the minor groove immediately upstream of the fork junction. This action is proposed to prevent the reannealing of t- and nt-strands and collapse of transcription bubble, thus stabilizing the RPo. The proposed mechanism of CarD action is strongly supported by the experimental evidence. First, CarD did not affect the conformation of RPo in the structure or alter the size of the transcription bubble during RPo formation on native promoters. Second, kinetic data showed that CarD increased the resistance of RPo to competitor challenge in *in vitro* assays on native promoters, although it had no effect on RPo assembled on artificial bubble templates (Davis et al., 2015). Finally, mutational analysis indicated that specific interaction between W86 and −12T of nt-strand plays a critical role in RPo stabilization (Bae et al., 2015a). Thus, transcription activation by CarD entails stabilizing the RPo, and prolonging its lifetime sufficient for successful initiation of RNA synthesis.

Analysis of CarD chromosomal distribution using ChipSeq revealed that CarD is associated with RNAP predominantly at promoter regions, co-localizing with σ^A^ (Stallings et al., 2009; Srivastava et al., 2013), suggesting that CarD dissociates during early stages of elongation. It's unclear what causes its dissociation. Since the CarD-RID is homologous to the NTD of Mfd, transcription coupled repair factor, which also binds to the β lobe-1, it is possible that CarD is displaced from elongation complex by Mfd. Also, because CarD stabilizes the RPo, it would be expected to negatively affect the rate of promoter escape. Additional biochemical experiments would be needed to address this hypothesis.

## Transcription inhibitors that target RNAP

Because bacterial RNAP performs essential functions in the cell, and because it differs sufficiently from eukaryotic RNAPs, it is an attractive target for antibiotics (for comprehensive review on the subject, see Ma et al., 2016). Currently, known drugs targeting RNAP can be divided into three groups based on their modes of action (Table 1): (i) those that disrupt RNAP interactions with DNA, RNA or NTPs; (ii) those that interfere with the movement of RNAP mobile elements during nucleotide addition cycle (NAC); and (iii) those that disrupt RNAP interactions with the housekeeping initiation factor, σ^70^. Although many of these drugs were discovered decades ago, and have been extensively characterized biochemically and genetically since then, it is only with the recent avalanche of structural data obtained for bacterial RNAP in complex many inhibitors that their mechanism of action began to be truly revealed at the molecular level (Murakami, 2015). Below, we summarize briefly the current understanding of how these drugs interact with RNAP from a structural point of view.

**Table 1:** **Small molecule inhibitors that target RNAP**.

**Classification**	**Name**	**Source**	**Binding site**	**Comment**	**References**
Inhibitors that disrupt	Rifamycins	*Amycolatopsis mediterranei*	β subunit active site cleft	High resistance spectrum	Campbell et al., 2001; Molodtsov et al., 2013
RNAP—DNA/RNA/NTPs interactions	Sorangicin	*Sorangium cellulosum*			Campbell et al., 2005
	GE23077	*Actinomadura* sp	i/i+1 NTP binding site	Low resistance spectrum but poor membrane permeability	Zhang et al., 2014
	GE-Rif	semi-synthetic	composite of Rif- and GE-binding sites		Zhang et al., 2014
	Microcin	*E. coli* AY25	2° channel (based on modeling)	High resistance spectrum, active against Gram(−) only	Adelman et al., 2004; Mukhopadhyay et al., 2004
	Myxopyronins	*Myxococcus fulvus*	β′ Switch-2	broad-spectrum antibacterial (Gram+ and Gram−)	Mukhopadhyay et al., 2008; Belogurov et al., 2009; Srivastava et al., 2011
	Corallopyronin	*Corallococcus coralloides*			
	Ripostatin	*Sorangium cellulosum*			
	Squaramides	synthetic			Buurman et al., 2012; Molodtsov et al., 2015
	Fidaxomicin	*Dactylosporangium aurantiacum*	β′ Switch-2 (σ^70/*S*^ R3.2)^*^	active against Gram (+)	Artsimovitch et al., 2012
	Lipiarmycin	*Actinoplanes deccanensis*			Tupin et al., 2010; Morichaud et al., 2016
Inhibitors that constrain the mobile elements of RNAP active center	Streptolydigin	*Streptomyces lydicus*	β′ BH, β′ TL, β fork loop-2 (downstream DNA)^*^	High resistance spectrum	Temiakov et al., 2005; Tuske et al., 2005; Vassylyev et al., 2007
	Salinamide	marine and soil *Streptomyces* spp	β′ BH hinge region (near 2°-channel), β′ F-loop, β-link, (β′ TL)^*^	Poor membrane permeability	Degen et al., 2014
	CBR compounds	chemical compound library	β element between fork loop 2 and β DII, β′ F-loop, β′ BH hinge region	Low resistance spectrum but tested only in *TolC* strain; cytotoxic	Artsimovitch et al., 2003, 2011; Malinen et al., 2012, 2014; Yuzenkova et al., 2013; Bae et al., 2015c; Feng et al., 2015
	Tagetitoxin	*Pseudomonas syringae* pv. *tagetis*	2°-channel; β′ catalytic loop and adjacent residues, β′ clip; β-link, β active site loop, Mg^2+^(II) (β′ TL)^*^	Low membrane permeability	Artsimovitch et al., 2011; Malinen et al., 2012; Yuzenkova et al., 2013
Inhibitors that disrupt RNAP-σ^70^ interactions	GKL003	chemical compound library	β′ clamp helices (CH)	High affinity (*K*_i_ ~6 nM) but low solubility and permeability	Ma et al., 2013; Yang et al., 2015a
	DSHS00507	drug-like compound library	β′ clamp helices (CH)	Effective against Gram-positive bacteria	
	SB series	compound library	undefined	IC_50_: 2~15 μM but low target specificity	André et al., 2004, 2006

* *Indicates possible auxiliary binding site*.

The first group comprises inhibitors that bind in the primary channel, the secondary channel, or to the β′ switch-2 region of RNAP. Rifamycins (RIF) and sorangicin bind in the primary channel near the active site, directly contacting β subunit, and sterically block the path of growing RNA beyond 2-3 nucleotides in length, effectively locking the abortive initiation complex at the promoter (Campbell et al., 2001, 2005; Molodtsov et al., 2013). GE23077 binds to the *i* and *i*+1 sites of the active center, immediately adjacent to the catalytic Mg^2+^ ion, and sterically occludes natural substrates from binding to these sites, inhibiting RNAP from initiating transcription *de novo* (Zhang et al., 2014). Using the structural information and modeling, a bipartite drug that binds to adjoining (but not overlapping) sites near the active center of RNAP was created by covalently linking GE and rifamycin SV (RIF derivative). The resulting compound, RifaGE-3, was active against both Rif^R^ and GE^R^ RNAPs (Zhang et al., 2014), suggesting that bipartite drugs could represent a new class of antibiotics to combat pathogenic bacteria that are increasingly drug-resistant. However, their large size and complexity may cause reduced permeability and increased cytotoxicity.

Microcin binds in the 2° channel and competitively prevents NTP uptake or binding, thereby inhibiting abortive initiation and elongation (Adelman et al., 2004; Mukhopadhyay et al., 2004). Compounds like myxopyronins, corallopyronin (Mukhopadhyay et al., 2008), ripostatin (Mukhopadhyay et al., 2008; Belogurov et al., 2009), and squaramides (Buurman et al., 2012; Molodtsov et al., 2015) bind to RNAP β′ switch-2 region that controls the hinged, swinging motion of β′ clamp, which in turn is responsible for the opening and closing of the primary channel (Srivastava et al., 2011). Binding of these compounds prevents the β′ clamp from opening, stabilizes the β′ clamp/switch regions in a partly closed/fully closed conformation, and prevents template DNA from reaching the active site. In particular, squaramide, in its co-crystal structure with RNAP, is shown to displace β′ switch-2 into the DNA binding main channel of RNAP (Molodtsov et al., 2015), which would interfere with proper placement of the melted template DNA (Bae et al., 2015b). Fidaxomicin and lipiarmycin (Tupin et al., 2010; Artsimovitch et al., 2012; Morichaud et al., 2016) are structurally closely related natural compounds that also bind to the β′ switch-2 region and prevent t-strand DNA from accessing the RNAP active-site cleft. Interestingly, the sensitivity of RNAP to lipiarmycin is aggravated in the presence of specific mutations in σ^70^ that are known to destabilize RPo (Morichaud et al., 2016). This result supports the assertion that lipiarmycin, and likely fidoxymicin, competes with t- strand DNA for the same binding site on RNAP β′ switch-2 region during RPo formation, and effectively inhibits promoter melting and RPo formation.

The second group comprises inhibitors that interact with, or bind near the catalytically important mobile elements of RNAP, β′ BH, β′ TL, β-link, and F-loop. These mobile elements are located in the immediate vicinity of the active site of RNAP, and are proposed to undergo conformational changes in concert with NAC (Malinen et al., 2012). Notably, the β′ TL alternates between “open” (unfolded) and “closed” (folded) states, while the adjacent β′ BH alternates between bent and straight conformations (Wang et al., 2006; Jovanovic et al., 2011). These structural changes are thought to accompany each NAC during transcription. In the “closed” conformation, β′ TL forms a three-helix bundle with β′ BH, and is directly contacted by the residues of F-loop and the β-link, which leads to further stabilization of the folded conformation of the β′ TL. NTP loading and catalysis occur in this state. In the “open” conformation, the three-helix bundle collapses: β′ BH bends toward RNA–DNA hybrid; β′ TL becomes unfolded. This state presumably permits RNA and DNA translocation following catalysis. Streptolydigin (Temiakov et al., 2005; Tuske et al., 2005; Vassylyev et al., 2007), salinamide (Degen et al., 2014), CBR (Artsimovitch et al., 2003, 2011; Malinen et al., 2012, 2014; Yuzenkova et al., 2013; Bae et al., 2015c; Feng et al., 2015), and, most likely targetitoxin (Artsimovitch et al., 2011; Malinen et al., 2012; Yuzenkova et al., 2013), are all inhibitors that bind RNAP and interact intimately with non-overlapping residues of β′ BH, β′ TL, β′ F-loop, and/or β-link in the active site milieu. Extensive structural and biochemical studies support the mechanistic model that these inhibitors stabilize an intermediate complex formed during NAC by immobilizing one or more mobile elements in a fixed conformation, thereby halting the iterative catalytic process. Binding sites of salinamide and CBR on *Eco* RNAP identified from analysis of X-ray co-crystal structures are consistent with their genetically-mapped binding sites. Interestingly, it was reported that two CBR^*R*^ mutations, P750 → L in β′ F-loop and F773 → V in β′ BH N-terminus, also conferred CBR dependence for cell growth (Bae et al., 2015c). One possible explanation for this observation is that the mutations made these mobile elements too flexible to support NAC, and that binding of CBR compensated for this defect. This interpretation would be consistent with the proposed mechanism of action of CBR.

The third group of inhibitors include compounds of GKL-, DSHS-, and SB-series, all derived from chemical compound libraries, that are predicted to directly inhibit RNAP-σ^70^ interaction. The SB-series compounds were discovered by screening the library using ELISA-based assay, and while they inhibited RNAP-σ^70^ association with IC_50_ ranging from 2 to 15 μM, many showed nonspecific binding to unrelated targets *in vivo* (André et al., 2004, 2006). GKL- and DSHS-series were screened *in silico* based on the strategy of structure-based drug design, and subsequently tested *in vitro* for validation (Ma et al., 2013; Yang et al., 2015a). As predicted by pharmacophore modeling, select compounds of GKL- and DSHS-series were shown to compete with σ^70^ for binding to RNAP core to form holoenzyme. More analysis and characterization is required to determine if these compounds can be further developed as potential antibacterial drugs. In theory, it should be possible to screen for compounds that inhibit σ *dissociation* from RPo using the same pharmacophore model (Ma et al., 2013), or one based on the RPo complex structure. An attractive target interface for such an inhibitor would be the RNA exit channel, where blocking of σ3.2 release would cause essentially the same inhibitory effect as RIF.

A survey of currently available RNAP-specific inhibitors reveals that for many lead compounds, the predicted *in vivo* effectiveness is often low due to their poor permeability, cytotoxicity, and broad resistance spectrum. Therefore, future drug designs will need to include strategies for incorporating effective delivery mechanisms, such as nanoparticles or functional conjugates that can be cleaved/unloaded inside the cell. Design for new drugs should also aim to improve solubility and reduce nonspecific, aggregate-forming properties of drugs associated with cytotoxicity. The construction of bipartite molecules, in principle, offers a highly promising approach to achieve increased potency and low resistance spectrum. It remains to be seen if, with the right combination of linked inhibitors and modifications, an effective bipartite drug can be constructed that is permeable with negligible toxicity. Finally, to design inhibitors of RNAP with narrow resistance spectrum, it is instructive to note that Sorangicin, which resembles RIF and binds to the same site on RNAP, exhibits a narrower resistance spectrum than RIF. This is attributed to its presumed greater conformational flexibility, enabling it to accommodate mutations in the RIF-binding pocket (Campbell et al., 2005). This suggests that a built-in structural flexibility of the compound may be an important factor in smart drug design.

## Concluding remarks

The last 15 years saw a remarkable progress in our understanding of the structure-function relationship of bacterial RNAP thanks to the advances in structural studies of this enzyme. It is hoped that in the near future, structural studies will continue to reveal fine details of transcription, especially of the events during formation of RPc, scrunching of RPinit and termination processes. Additionally, an exciting new direction in RNAP research is emerging with the advent of high throughput sequencing and screening techniques, which will help to shed new light on the function of RNAP in the context of true physiological environment.

## Author contributions

JL and SB equally contributed to the preparation and writing of the manuscript.

## Funding

Research in SB lab is supported by the Department of Cell Biology and the Graduate School of Biomedical Studies at Rowan University.

### Conflict of interest statement

The authors declare that the research was conducted in the absence of any commercial or financial relationships that could be construed as a potential conflict of interest.

## References

[B1] Adelman K. Yuzenkova J. La Porta A. Zenkin N. Lee J. Lis J. T.. (2004). Molecular mechanism of transcription inhibition by peptide antibiotic Microcin J25. Mol. Cell 14, 753–762. 10.1016/j.molcel.2004.05.017 15200953

[B2] André E. Bastide L. Michaux-Charachon S. Gouby A. Villain-Guillot P. Latouche J.. (2006). Novel synthetic molecules targeting the bacterial RNA polymerase assembly. J. Antimicrob. Chemother. 57, 245–251. 10.1093/jac/dki426 16373430

[B3] André E. Bastide L. Villain-Guillot P. Latouche J. Rouby J. Leonetti J. P. (2004). A multiwell assay to isolate compounds inhibiting the assembly of the prokaryotic RNA polymerase. Assay Drug Dev. Technol. 2, 629–635. 10.1089/adt.2004.2.629 15674021

[B4] Artsimovitch I. Chu C. Lynch A. S. Landick R. (2003). A new class of bacterial RNA polymerase inhibitor affects nucleotide addition. Science 302, 650–654. 10.1126/science.1087526 14576436

[B5] Artsimovitch I. Seddon J. Sears P. (2012). Fidaxomicin is an inhibitor of the initiation of bacterial RNA synthesis. Clin. Infect. Dis. 55(Suppl. 2), S127–S131. 10.1093/cid/cis358 22752861 PMC3388026

[B6] Artsimovitch I. Svetlov V. Nemetski S. M. Epshtein V. Cardozo T. Nudler E. (2011). Tagetitoxin inhibits RNA polymerase through trapping of the trigger loop. J. Biol. Chem. 286, 40395–40400. 10.1074/jbc.M111.300889 21976682 PMC3220573

[B7] Bae B. Chen J. Davis E. Leon K. Darst S. A. Campbell E. A. (2015a). CarD uses a minor groove wedge mechanism to stabilize the RNA polymerase open promoter complex. Elife 4:e08505. 10.7554/eLife.08505 26349034 PMC4593161

[B8] Bae B. Davis E. Brown D. Campbell E. A. Wigneshweraraj S. Darst S. A. (2013). Phage T7 Gp2 inhibition of *Escherichia coli* RNA polymerase involves misappropriation of sigma70 domain 1.1. Proc. Natl. Acad. Sci. U.S.A. 110, 19772–19777. 10.1073/pnas.1314576110 24218560 PMC3856789

[B9] Bae B. Feklistov A. Lass-Napiorkowska A. Landick R. Darst S. A. (2015b). Structure of a bacterial RNA polymerase holoenzyme open promoter complex. Elife 4:e08504. 10.7554/eLife.08504 26349032 PMC4593229

[B10] Bae B. Nayak D. Ray A. Mustaev A. Landick R. Darst S. A. (2015c). CBR antimicrobials inhibit RNA polymerase via at least two bridge-helix cap-mediated effects on nucleotide addition. Proc. Natl. Acad. Sci. U.S.A. 112, E4178–E4187. 10.1073/pnas.1502368112 26195788 PMC4534225

[B11] Basu R. S. Warner B. A. Molodtsov V. Pupov D. Esyunina D. Fernández-Tornero C.. (2014). Structural basis of transcription initiation by bacterial RNA polymerase holoenzyme. J. Biol. Chem. 289, 24549–24559. 10.1074/jbc.M114.584037 24973216 PMC4148879

[B12] Belogurov G. A. Artsimovitch I. (2015). Regulation of Transcript Elongation. Annu. Rev. Microbiol. 69, 49–69. 10.1146/annurev-micro-091014-104047 26132790 PMC4674076

[B13] Belogurov G. A. Vassylyeva M. N. Sevostyanova A. Appleman J. R. Xiang A. X. Lira R.. (2009). Transcription inactivation through local refolding of the RNA polymerase structure. Nature 457, 332–335. 10.1038/nature07510 18946472 PMC2628454

[B14] Benoff B. Yang H. Lawson C. L. Parkinson G. Liu J. Blatter E.. (2002). Structural basis of transcription activation: the CAP-alpha CTD-DNA complex. Science 297, 1562–1566. 10.1126/science.1076376 12202833

[B15] Borukhov S. Nudler E. (2003). RNA polymerase holoenzyme: structure, function and biological implications. Curr. Opin. Microbiol. 6, 93–100. 10.1016/S1369-5274(03)00036-5 12732296

[B16] Borukhov S. Nudler E. (2008). RNA polymerase: the vehicle of transcription. Trends Microbiol. 16, 126–134. 10.1016/j.tim.2007.12.006 18280161

[B17] Borukhov S. Sagitov V. Josaitis C. A. Gourse R. L. Goldfarb A. (1993). Two modes of transcription initiation *in vitro* at the rrnB P1 promoter of *Escherichia coli*. J. Biol. Chem. 268, 23477–23482. 8226874

[B18] Buurman E. T. Foulk M. A. Gao N. Laganas V. A. McKinney D. C. Moustakas D. T.. (2012). Novel rapidly diversifiable antimicrobial RNA polymerase switch region inhibitors with confirmed mode of action in *Haemophilus influenzae*. J. Bacteriol. 194, 5504–5512. 10.1128/JB.01103-12 22843845 PMC3458661

[B19] Campbell E. A. Korzheva N. Mustaev A. Murakami K. Nair S. Goldfarb A.. (2001). Structural mechanism for rifampicin inhibition of bacterial rna polymerase. Cell 104, 901–912. 10.1016/S0092-8674(01)00286-0 11290327

[B20] Campbell E. A. Muzzin O. Chlenov M. Sun J. L. Olson C. A. Weinman O.. (2002). Structure of the bacterial RNA polymerase promoter specificity sigma subunit. Mol. Cell 9, 527–539. 10.1016/S1097-2765(02)00470-7 11931761

[B21] Campbell E. A. Pavlova O. Zenkin N. Leon F. Irschik H. Jansen R.. (2005). Structural, functional, and genetic analysis of sorangicin inhibition of bacterial RNA polymerase. EMBO J. 24, 674–682. 10.1038/sj.emboj.7600499 15692574 PMC549610

[B22] Chen Y. F. Helmann J. D. (1997). DNA-melting at the Bacillus subtilis flagellin promoter nucleates near -10 and expands unidirectionally. J. Mol. Biol. 267, 47–59. 10.1006/jmbi.1996.0853 9096206

[B23] Daniels D. Zuber P. Losick R. (1990). Two amino acids in an RNA polymerase sigma factor involved in the recognition of adjacent base pairs in the -10 region of a cognate promoter. Proc. Natl. Acad. Sci. U.S.A. 87, 8075–8079. 10.1073/pnas.87.20.8075 2122453 PMC54895

[B24] Davis E. Chen J. Leon K. Darst S. A. Campbell E. A. (2015). Mycobacterial RNA polymerase forms unstable open promoter complexes that are stabilized by CarD. Nucleic Acids Res. 43, 433–445. 10.1093/nar/gku1231 25510492 PMC4288152

[B25] Decker K. B. Hinton D. M. (2013). Transcription regulation at the core: similarities among bacterial, archaeal, and eukaryotic RNA polymerases. Annu. Rev. Microbiol. 67, 113–139. 10.1146/annurev-micro-092412-155756 23768203

[B26] Degen D. Feng Y. Zhang Y. Ebright K. Y. Ebright Y. W. Gigliotti M.. (2014). Transcription inhibition by the depsipeptide antibiotic salinamide A. Elife 3:e02451. 10.7554/eLife.02451 24843001 PMC4029172

[B27] Feklistov A. (2013). RNA polymerase: in search of promoters. Ann. N.Y. Acad. Sci. 1293, 25–32. 10.1111/nyas.12197 23855603 PMC3740395

[B28] Feklistov A. Barinova N. Sevostyanova A. Heyduk E. Bass I. Vvedenskaya I.. (2006). A basal promoter element recognized by free RNA polymerase sigma subunit determines promoter recognition by RNA polymerase holoenzyme. Mol. Cell 23, 97–107. 10.1016/j.molcel.2006.06.010 16798040

[B29] Feklistov A. Darst S. A. (2011). Structural basis for promoter-10 element recognition by the bacterial RNA polymerase sigma subunit. Cell 147, 1257–1269. 10.1016/j.cell.2011.10.041 22136875 PMC3245737

[B30] Feklístov A. Sharon B. D. Darst S. A. Gross C. A. (2014). Bacterial sigma factors: a historical, structural, and genomic perspective. Annu. Rev. Microbiol. 68, 357–376. 10.1146/annurev-micro-092412-155737 25002089

[B31] Feng Y. Degen D. Wang X. Gigliotti M. Liu S. Zhang Y.. (2015). tructural Basis of Transcription Inhibition by CBR Hydroxamidines and CBR Pyrazoles. Structure 23, 1470–1481. 10.1016/j.str.2015.06.009 26190576 PMC4526357

[B32] Feng Y. Zhang Y. Ebright R. H. (2016). Structural basis of transcription activation. Science 352, 1330–1333. 10.1126/science.aaf4417 27284196 PMC4905602

[B33] Goldman S. R. Nair N. U. Wells C. D. Nickels B. E. Hochschild A. (2015). The primary sigma factor in Escherichia coli can access the transcription elongation complex from solution *in vivo*. Elife 4:e10514. 10.7554/eLife.10514 26371553 PMC4604602

[B34] Gruber T. M. Gross C. A. (2003). Multiple sigma subunits and the partitioning of bacterial transcription space. Annu. Rev. Microbiol. 57, 441–466. 10.1146/annurev.micro.57.030502.090913 14527287

[B35] Gruber T. M. Markov D. Sharp M. M. Young B. A. Lu C. Z. Zhong H. J.. (2001). Binding of the initiation factor sigma to core RNA polymerase is a multistep process. Mol. Cell 8, 21–31. 10.1016/S1097-2765(01)00292-1 11511357

[B36] Gulten G. Sacchettini J. C. (2013). Structure of the Mtb CarD/RNAP beta-lobes complex reveals the molecular basis of interaction and presents a distinct DNA-binding domain for Mtb CarD. Structure 21, 1859–1869. 10.1016/j.str.2013.08.014 24055315 PMC3894638

[B37] Haugen S. P. Berkmen M. B. Ross W. Gaal T. Ward C. Gourse R. L. (2006). rRNA promoter regulation by nonoptimal binding of sigma region 1.2: an additional recognition element for RNA polymerase. Cell 125, 1069–1082. 10.1016/j.cell.2006.04.034 16777598

[B38] Haugen S. P. Ross W. Gourse R. L. (2008). Advances in bacterial promoter recognition and its control by factors that do not bind DNA. Nat. Rev. Microbiol. 6, 507–519. 10.1038/nrmicro1912 18521075 PMC3700611

[B39] Hook-Barnard I. G. Hinton D. M. (2007). Transcription initiation by mix and match elements: flexibility for polymerase binding to bacterial promoters. Gene Regul. Syst. Bio. 1, 275–293. 19119427 PMC2613000

[B40] Hudson B. P. Quispe J. Lara-González S. Kim Y. Berman H. M. Arnold E.. (2009). Three-dimensional EM structure of an intact activator-dependent transcription initiation complex. Proc. Natl. Acad. Sci. U.S.A. 106, 19830–19835. 10.1073/pnas.0908782106 19903881 PMC2775702

[B41] Imashimizu M. Shimamoto N. Oshima T. Kashlev M. (2014). Transcription elongation: heterogeneous tracking of RNA polymerase and its biological implications. Transcription 5:e28285. 10.4161/trns.28285 25764114 PMC4214235

[B42] Jovanovic M. Burrows P. C. Bose D. Cámara B. Wiesler S. Zhang X.. (2011). Activity map of the Escherichia coli RNA polymerase bridge helix. J. Biol. Chem. 286, 14469–14479. 10.1074/jbc.M110.212902 21357417 PMC3077646

[B43] Kamarthapu V. Epshtein V. Benjamin B. Proshkin S. Mironov A. Cashel M.. (2016). ppGpp couples transcription to DNA repair in *E. coli*. Science 352, 993–996. 10.1126/science.aad6945 27199428 PMC4917784

[B44] Kapanidis A. N. Margeat E. Ho S. O. Kortkhonjia E. Weiss S. Ebright R. H. (2006). Initial transcription by RNA polymerase proceeds through a DNA-scrunching mechanism. Science 314, 1144–1147. 10.1126/science.1131399 17110578 PMC2754788

[B45] Keilty S. Rosenberg M. (1987). Constitutive function of a positively regulated promoter reveals new sequences essential for activity. J. Biol. Chem. 262, 6389–6395. 3032964

[B46] Kireeva M. Kashlev M. Burton Z. F. (2010). Translocation by multi-subunit RNA polymerases. Biochim. Biophys. Acta 1799, 389–401. 10.1016/j.bbagrm.2010.01.007 20097318

[B47] Kriner M. A. Sevostyanova A. Groisman E. A. (2016). Learning from the Leaders: gene regulation by the transcription termination factor Rho. Trends Biochem. Sci. 41, 690–699. 10.1016/j.tibs.2016.05.012 27325240 PMC4967001

[B48] Kulbachinskiy A. Mustaev A. (2006). Region 3.2 of the sigma subunit contributes to the binding of the 3′-initiating nucleotide in the RNA polymerase active center and facilitates promoter clearance during initiation. J. Biol. Chem. 281, 18273–18276. 10.1074/jbc.C600060200 16690607

[B49] Landick R. (2006). The regulatory roles and mechanism of transcriptional pausing. Biochem. Soc. Trans. 34(Pt 6), 1062–1066. 10.1042/BST0341062 17073751

[B50] Lawson C. L. Swigon D. Murakami K. S. Darst S. A. Berman H. M. Ebright R. H. (2004). Catabolite activator protein: DNA binding and transcription activation. Curr. Opin. Struct. Biol. 14, 10–20. 10.1016/j.sbi.2004.01.012 15102444 PMC2765107

[B51] Lee D. J. Minchin S. D. Busby S. J. (2012). Activating transcription in bacteria. Annu. Rev. Microbiol. 66, 125–152. 10.1146/annurev-micro-092611-150012 22726217

[B52] Lim H. M. Lee H. J. Roy S. Adhya S. (2001). A “master” in base unpairing during isomerization of a promoter upon RNA polymerase binding. Proc. Natl. Acad. Sci. U.S.A. 98, 14849–14852. 10.1073/pnas.261517398 11734629 PMC64947

[B53] Liu B. Zuo Y. Steitz T. A. (2015). Structural basis for transcription reactivation by RapA. Proc. Natl. Acad. Sci. U.S.A. 112, 2006–2010. 10.1073/pnas.1417152112 25646438 PMC4343176

[B54] Liu B. Zuo Y. Steitz T. A. (2016). Structures of *E. coli* sigmaS-transcription initiation complexes provide new insights into polymerase mechanism. Proc. Natl. Acad. Sci. U.S.A. 113, 4051–4056. 10.1073/pnas.1520555113 27035955 PMC4839411

[B55] Liu M. Tolstorukov M. Zhurkin V. Garges S. Adhya S. (2004). A mutant spacer sequence between −35 and −10 elements makes the Plac promoter hyperactive and cAMP receptor protein-independent. Proc. Natl. Acad. Sci. U.S.A. 101, 6911–6916. 10.1073/pnas.0401929101 15118087 PMC406441

[B56] Ma C. Yang X. Kandemir H. Mielczarek M. Johnston E. B. Griffith R.. (2013). Inhibitors of bacterial transcription initiation complex formation. ACS Chem. Biol. 8, 1972–1980. 10.1021/cb400231p 23751807

[B57] Ma C. Yang X. Lewis P. J. (2016). Bacterial transcription as a target for antibacterial drug development. Microbiol. Mol. Biol. Rev. 80, 139–160. 10.1128/MMBR.00055-15 26764017 PMC4771368

[B58] Maeda H. Fujita N. Ishihama A. (2000). Competition among seven *Escherichia coli* sigma subunits: relative binding affinities to the core RNA polymerase. Nucleic Acids Res. 28, 3497–3503. 10.1093/nar/28.18.3497 10982868 PMC110723

[B59] Malinen A. M. Nandymazumdar M. Turtola M. Malmi H. Grocholski T. Artsimovitch I.. (2014). CBR antimicrobials alter coupling between the bridge helix and the beta subunit in RNA polymerase. Nat. Commun. 5, 3408. 10.1038/ncomms4408 24598909 PMC3959191

[B60] Malinen A. M. Turtola M. Parthiban M. Vainonen L. Johnson M. S. Belogurov G. A. (2012). Active site opening and closure control translocation of multisubunit RNA polymerase. Nucleic Acids Res. 40, 7442–7451. 10.1093/nar/gks383 22570421 PMC3424550

[B61] Mekler V. Kortkhonjia E. Mukhopadhyay J. Knight J. Revyakin A. Kapanidis A. N.. (2002). Structural organization of bacterial RNA polymerase holoenzyme and the RNA polymerase-promoter open complex. Cell 108, 599–614. 10.1016/S0092-8674(02)00667-0 11893332

[B62] Molodtsov V. Fleming P. R. Eyermann C. J. Ferguson A. D. Foulk M. A. McKinney D. C.. (2015). X-ray crystal structures of *Escherichia coli* RNA polymerase with switch region binding inhibitors enable rational design of squaramides with an improved fraction unbound to human plasma protein. J. Med. Chem. 58, 3156–3171. 10.1021/acs.jmedchem.5b00050 25798859 PMC4658208

[B63] Molodtsov V. Nawarathne I. N. Scharf N. T. Kirchhoff P. D. Showalter H. D. Garcia G. A.. (2013). X-ray crystal structures of the *Escherichia coli* RNA polymerase in complex with benzoxazinorifamycins. J. Med. Chem. 56, 4758–4763. 10.1021/jm4004889 23679862 PMC3745299

[B64] Morichaud Z. Chaloin L. Brodolin K. (2016). Regions 1.2 and 3.2 of the RNA Polymerase sigma subunit promote DNA melting and attenuate action of the antibiotic lipiarmycin. J. Mol. Biol. 428, 463–476. 10.1016/j.jmb.2015.12.017 26724534

[B65] Mukhopadhyay J. Das K. Ismail S. Koppstein D. Jang M. Hudson B.. (2008). The RNA polymerase “switch region” is a target for inhibitors. Cell 135, 295–307. 10.1016/j.cell.2008.09.033 18957204 PMC2580802

[B66] Mukhopadhyay J. Sineva E. Knight J. Levy R. M. Ebright R. H. (2004). Antibacterial peptide microcin J25 inhibits transcription by binding within and obstructing the RNA polymerase secondary channel. Mol. Cell 14, 739–751. 10.1016/j.molcel.2004.06.010 15200952 PMC2754415

[B67] Murakami K. S. (2013). X-ray crystal structure of *Escherichia coli* RNA polymerase sigma70 holoenzyme. J. Biol. Chem. 288, 9126–9134. 10.1074/jbc.M112.430900 23389035 PMC3610985

[B68] Murakami K. S. (2015). Structural biology of bacterial RNA polymerase. Biomolecules 5, 848–864. 10.3390/biom5020848 25970587 PMC4496699

[B69] Murakami K. S. Darst S. A. (2003). Bacterial RNA polymerases: the wholo story. Curr. Opin. Struct. Biol. 13, 31–39. 10.1016/S0959-440X(02)00005-2 12581657

[B70] Murakami K. S. Masuda S. Campbell E. A. Muzzin O. Darst S. A. (2002a). Structural basis of transcription initiation: an RNA polymerase holoenzyme-DNA complex. Science 296, 1285–1290. 10.1126/science.1069595 12016307

[B71] Murakami K. S. Masuda S. Darst S. A. (2002b). Structural basis of transcription initiation: RNA polymerase holoenzyme at 4 A resolution. Science 296, 1280–1284. 10.1126/science.1069594 12016306

[B72] Niu W. Kim Y. Tau G. Heyduk T. Ebright R. H. (1996). Transcription activation at class II CAP-dependent promoters: two interactions between CAP and RNA polymerase. Cell 87, 1123–1134. 10.1016/S0092-8674(00)81806-1 8978616 PMC4430116

[B73] Nudler E. (2009). RNA polymerase active center: the molecular engine of transcription. Annu. Rev. Biochem. 78, 335–361. 10.1146/annurev.biochem.76.052705.164655 19489723 PMC2929140

[B74] Nudler E. (2012). RNA polymerase backtracking in gene regulation and genome instability. Cell 149, 1438–1445. 10.1016/j.cell.2012.06.003 22726433 PMC3815583

[B75] Patrick M. Dennis P. P. Ehrenberg M. Bremer H. (2015). Free RNA polymerase in *Escherichia coli*. Biochimie 119, 80–91. 10.1016/j.biochi.2015.10.015 26482806

[B76] Perdue S. A. Roberts J. W. (2011). Sigma(70)-dependent transcription pausing in *Escherichia coli*. J. Mol. Biol. 412, 782–792. 10.1016/j.jmb.2011.02.011 21316374

[B77] Pupov D. Kuzin I. Bass I. Kulbachinskiy A. (2014). Distinct functions of the RNA polymerase sigma subunit region 3.2 in RNA priming and promoter escape. Nucleic Acids Res. 42, 4494–4504. 10.1093/nar/gkt1384 24452800 PMC3985618

[B78] Revyakin A. Liu C. Ebright R. H. Strick T. R. (2006). Abortive initiation and productive initiation by RNA polymerase involve DNA scrunching. Science 314, 1139–1143. 10.1126/science.1131398 17110577 PMC2754787

[B79] Rhodius V. A. Busby S. J. (2000). Transcription activation by the Escherichia coli cyclic AMP receptor protein: determinants within activating region 3. J. Mol. Biol. 299, 295–310. 10.1006/jmbi.2000.3736 10860739

[B80] Rhodius V. A. West D. M. Webster C. L. Busby S. J. Savery N. J. (1997). Transcription activation at class II CRP-dependent promoters: the role of different activating regions. Nucleic Acids Res. 25, 326–332. 10.1093/nar/25.2.326 9016561 PMC146447

[B81] Robb N. C. Cordes T. Hwang L. C. Gryte K. Duchi D. Craggs T. D.. (2013). The transcription bubble of the RNA polymerase-promoter open complex exhibits conformational heterogeneity and millisecond-scale dynamics: implications for transcription start-site selection. J. Mol. Biol. 425, 875–885. 10.1016/j.jmb.2012.12.015 23274143 PMC3783996

[B82] Roberts J. Park J. S. (2004). Mfd, the bacterial transcription repair coupling factor: translocation, repair and termination. Curr. Opin. Microbiol. 7, 120–125. 10.1016/j.mib.2004.02.014 15063847

[B83] Ross W. Ernst A. Gourse R. L. (2001). Fine structure of *E. coli* RNA polymerase-promoter interactions: alpha subunit binding to the UP element minor groove. Genes Dev. 15, 491–506. 10.1101/gad.870001 11238372 PMC312649

[B84] Ross W. Gosink K. K. Salomon J. Igarashi K. Zou C. Ishihama A.. (1993). A third recognition element in bacterial promoters: DNA binding by the alpha subunit of RNA polymerase. Science 262, 1407–1413. 10.1126/science.8248780 8248780

[B85] Ross W. Gourse R. L. (2009). Analysis of RNA polymerase-promoter complex formation. Methods 47, 13–24. 10.1016/j.ymeth.2008.10.018 18952176 PMC2633133

[B86] Roy S. Garges S. Adhya S. (1998). Activation and repression of transcription by differential contact: two sides of a coin. J. Biol. Chem. 273, 14059–14062. 10.1074/jbc.273.23.14059 9603899

[B87] Saecker R. M. Record M. T. Jr. Dehaseth P. L. (2011). Mechanism of bacterial transcription initiation: RNA polymerase - promoter binding, isomerization to initiation-competent open complexes, and initiation of RNA synthesis. J. Mol. Biol. 412, 754–771. 10.1016/j.jmb.2011.01.018 21371479 PMC3440003

[B88] Samanta S. Martin C. T. (2013). Insights into the mechanism of initial transcription in Escherichia coli RNA polymerase. J. Biol. Chem. 288, 31993–32003. 10.1074/jbc.M113.497669 24047893 PMC3814795

[B89] Sarkar P. Sardesai A. A. Murakami K. S. Chatterji D. (2013). Inactivation of the bacterial RNA polymerase due to acquisition of secondary structure by the omega subunit. J. Biol. Chem. 288, 25076–25087. 10.1074/jbc.M113.468520 23843456 PMC3757172

[B90] Sen R. Nagai H. Hernandez V. J. Shimamoto N. (1998). Reduction in abortive transcription from the lambdaPR promoter by mutations in region 3 of the sigma70 subunit of *Escherichia coli* RNA polymerase. J. Biol. Chem. 273, 9872–9877. 10.1074/jbc.273.16.9872 9545328

[B91] Sengupta S. Prajapati R. K. Mukhopadhyay J. (2015). Promoter Escape with bacterial two-component sigma factor suggests retention of sigma region two in the elongation complex. J. Biol. Chem. 290, 28575–28583. 10.1074/jbc.M115.666008 26400263 PMC4653711

[B92] Sharp M. M. Chan C. L. Lu C. Z. Marr M. T. Nechaev S. Merritt E. W.. (1999). The interface of sigma with core RNA polymerase is extensive, conserved, and functionally specialized. Genes Dev. 13, 3015–3026. 10.1101/gad.13.22.3015 10580008 PMC317155

[B93] Shorenstein R. G. Losick R. (1973). Purification and properties of the sigma subunit of ribonucleic acid polymerase from vegetative *Bacillus subtilis*. J. Biol. Chem. 248, 6163–6169. 4199260

[B94] Shultzaberger R. K. Chen Z. Lewis K. A. Schneider T. D. (2007). Anatomy of *Escherichia coli* sigma70 promoters. Nucleic Acids Res. 35, 771–788. 10.1093/nar/gkl956 17189297 PMC1807945

[B95] Srivastava A. Talaue M. Liu S. Degen D. Ebright R. Y. Sineva E.. (2011). New target for inhibition of bacterial RNA polymerase: ‘switch region.’ Curr. Opin. Microbiol. 14, 532–543. 10.1016/j.mib.2011.07.030 21862392 PMC3196380

[B96] Srivastava D. B. Leon K. Osmundson J. Garner A. L. Weiss L. A. Westblade L. F.. (2013). Structure and function of CarD, an essential mycobacterial transcription factor. Proc. Natl. Acad. Sci. U.S.A. 110, 12619–12624. 10.1073/pnas.1308270110 23858468 PMC3732983

[B97] Stallings C. L. Stephanou N. C. Chu L. Hochschild A. Nickels B. E. Glickman M. S. (2009). CarD is an essential regulator of rRNA transcription required for Mycobacterium tuberculosis persistence. Cell 138, 146–159. 10.1016/j.cell.2009.04.041 19596241 PMC2756155

[B98] Svetlov V. Nudler E. (2009). Macromolecular micromovements: how RNA polymerase translocates. Curr. Opin. Struct. Biol. 19, 701–707. 10.1016/j.sbi.2009.10.002 19889534 PMC3814128

[B99] Temiakov D. Zenkin N. Vassylyeva M. N. Perederina A. Tahirov T. H. Kashkina E.. (2005). Structural basis of transcription inhibition by antibiotic streptolydigin. Mol. Cell 19, 655–666. 10.1016/j.molcel.2005.07.020 16167380

[B100] Tupin A. Gualtieri M. Leonetti J. P. Brodolin K. (2010). The transcription inhibitor lipiarmycin blocks DNA fitting into the RNA polymerase catalytic site. EMBO J. 29, 2527–2537. 10.1038/emboj.2010.135 20562828 PMC2928680

[B101] Tuske S. Sarafianos S. G. Wang X. Hudson B. Sineva E. Mukhopadhyay J.. (2005). Inhibition of bacterial RNA polymerase by streptolydigin: stabilization of a straight-bridge-helix active-center conformation. Cell 122, 541–552. 10.1016/j.cell.2005.07.017 16122422 PMC2754413

[B102] Vassylyev D. G. Sekine S. Laptenko O. Lee J. Vassylyeva M. N. Borukhov S.. (2002). Crystal structure of a bacterial RNA polymerase holoenzyme at 2.6 A resolution. Nature 417, 712–719. 10.1038/nature752 12000971

[B103] Vassylyev D. G. Vassylyeva M. N. Zhang J. Palangat M. Artsimovitch I. Landick R. (2007). Structural basis for substrate loading in bacterial RNA polymerase. Nature 448, 163–168. 10.1038/nature05931 17581591

[B104] Vvedenskaya I. O. Vahedian-Movahed H. Zhang Y. Taylor D. M. Ebright R. H. Nickels B. E. (2016). Interactions between RNA polymerase and the core recognition element are a determinant of transcription start site selection. Proc. Natl. Acad. Sci. U.S.A. 113, E2899–E2905. 10.1073/pnas.1603271113 27162333 PMC4889395

[B105] Vvedenskaya I. O. Zhang Y. Goldman S. R. Valenti A. Visone V. Taylor D. M.. (2015). Massively systematic transcript end readout, “MASTER”: transcription start site selection, transcriptional slippage, and transcript yields. Mol. Cell 60, 953–965. 10.1016/j.molcel.2015.10.029 26626484 PMC4688149

[B106] Wang D. Bushnell D. A. Westover K. D. Kaplan C. D. Kornberg R. D. (2006). Structural basis of transcription: role of the trigger loop in substrate specificity and catalysis. Cell 127, 941–954. 10.1016/j.cell.2006.11.023 17129781 PMC1876690

[B107] Winkelman J. T. Chandrangsu P. Ross W. Gourse R. L. (2016a). Open complex scrunching before nucleotide addition accounts for the unusual transcription start site of *E. coli* ribosomal RNA promoters. Proc. Natl. Acad. Sci. U.S.A. 113, E1787–E1795. 10.1073/pnas.1522159113 26976590 PMC4822585

[B108] Winkelman J. T. Vvedenskaya I. O. Zhang Y. Zhang Y. Bird J. G. Taylor D. M.. (2016b). Multiplexed protein-DNA cross-linking: Scrunching in transcription start site selection. Science 351, 1090–1093. 10.1126/science.aad6881 26941320 PMC4797950

[B109] Winkelman J. T. Winkelman B. T. Boyce J. Maloney M. F. Chen A. Y. Ross W.. (2015). Crosslink mapping at amino acid-base resolution reveals the path of scrunched DNA in initial transcribing complexes. Mol. Cell 59, 768–780. 10.1016/j.molcel.2015.06.037 26257284 PMC4561013

[B110] Yang X. Ma C. Lewis P. J. (2015a). Identification of inhibitors of bacterial RNA polymerase. Methods 86, 45–50. 10.1016/j.ymeth.2015.05.005 25976836

[B111] Yang Y. Darbari V. C. Zhang N. Lu D. Glyde R. Wang Y. P.. (2015b). TRANSCRIPTION. Structures of the RNA polymerase-sigma54 reveal new and conserved regulatory strategies. Science 349, 882–885. 10.1126/science.aab1478 26293966 PMC4681505

[B112] Yarnell W. S. Roberts J. W. (1999). Mechanism of intrinsic transcription termination and antitermination. Science 284, 611–615. 10.1126/science.284.5414.611 10213678

[B113] Yuzenkova Y. Roghanian M. Bochkareva A. Zenkin N. (2013). Tagetitoxin inhibits transcription by stabilizing pre-translocated state of the elongation complex. Nucleic Acids Res. 41, 9257–9265. 10.1093/nar/gkt708 23935117 PMC3814378

[B114] Yuzenkova Y. Tadigotla V. R. Severinov K. Zenkin N. (2011). A new basal promoter element recognized by RNA polymerase core enzyme. EMBO J. 30, 3766–3775. 10.1038/emboj.2011.252 21792175 PMC3173786

[B115] Zhang G. Campbell E. A. Minakhin L. Richter C. Severinov K. Darst S. A. (1999). Crystal structure of *Thermus aquaticus* core RNA polymerase at 3.3 A resolution. Cell 98, 811–824. 10.1016/S0092-8674(00)81515-9 10499798

[B116] Zhang Y. Degen D. Ho M. X. Sineva E. Ebright K. Y. Ebright Y. W.. (2014). GE23077 binds to the RNA polymerase ‘i’ and ‘i+1’ sites and prevents the binding of initiating nucleotides. Elife 3:e02450. 10.7554/eLife.02450 24755292 PMC3994528

[B117] Zhang Y. Feng Y. Chatterjee S. Tuske S. Ho M. X. Arnold E.. (2012). Structural basis of transcription initiation. Science 338, 1076–1080. 10.1126/science.1227786 23086998 PMC3593053

[B118] Zuo Y. Steitz T. A. (2015). Crystal structures of the *E. coli* transcription initiation complexes with a complete bubble. Mol. Cell 58, 534–540. 10.1016/j.molcel.2015.03.010 25866247 PMC5567806

[B119] Zuo Y. Wang Y. Steitz T. A. (2013). The mechanism of *E. coli* RNA polymerase regulation by ppGpp is suggested by the structure of their complex. Mol. Cell 50, 430–436. 10.1016/j.molcel.2013.03.020 23623685 PMC3677725

